# Is sex a proxy for mechanical variables during an upper limb repetitive movement task? An investigation of the effects of sex and of anthropometric load on muscle fatigue

**DOI:** 10.1186/s13293-020-00336-1

**Published:** 2020-10-30

**Authors:** Matthew Slopecki, Karen Messing, Julie N. Côté

**Affiliations:** 1 Department of Kinesiology and Physical Education, McGill University, 475 Pine Ave W, Montreal, Quebec, H2W 1S4 Canada; 2 CINBIOSE Research Centre, Université du Québec à Montréal (UQAM), 8888, Succ. Centre-ville, Montreal, Quebec, H3C 3P8 Canada

**Keywords:** Fatigue, Sex differences, Upper limb, Shoulder, Musculoskeletal disorders, Neck/shoulder, Repetitive work, Anthropometrics

## Abstract

**Background:**

Women report more work-related pain and neck/shoulder musculoskeletal disorders than men. For the same absolute workload, due to lower strength, females generally work at a higher relative intensity, which could induce more fatigue. However, the arm’s anthropometric load (AL) of men is higher. Therefore, simply lifting their arm could be more fatiguing. Sex as a variable is formed of many constructs, and analyses can become muddied by their differing responses to fatigue. No studies have considered AL, when comparing how fatigue affects men and women. The purpose was to determine if including the arm’s AL in the statistical analysis would impact findings of sex-specific effects of shoulder fatigue on muscle EMG.

**Methods:**

Fifty-five (29m/26f) participants completed a repetitive pointing task (RPT) at shoulder height until they reported fatigue of 8+ on the BORG CR-10 scale. Muscle activities were measured using surface electrodes placed over the anterior deltoid (AD) and upper trapezius (UT) muscles. Muscle activity amplitude was quantified using root mean square (RMS). First- and last-minute data were used to assess change from no-fatigue (NF) to fatigue-terminal (FT) conditions. AL was calculated using sex-specific body parameter equations. General estimating equations (GEE) were used to determine the effects of sex and fatigue on RMS values, while including AL in the GEE.

**Results:**

There was no sex difference in time to reach fatigue. A significant main effect of sex on RMS was observed (*χ*^2^(1) = 4.17, *p* = 0.04) when including AL as a covariate. Females displayed a significantly higher percentage change in AD RMS from NF to FT, compared to males (*p* = 0.03), when AL was included in the GEE. No sex differences in UT were observed.

**Conclusions:**

This sex difference emerged when AL was included as a covariate, suggesting that sex-associated anthropometric differences may contribute to sex differences in the fatigue response. Differences in the impact of AL on AD compared to UT could be explained by differences in their respective mechanical roles or muscle fiber content. Anthropometrics may be useful to include as covariates in future research to separate individual anthropometric differences from sex differences.

## Background

Compared to men, women report more work-related pain and have a greater incidence of musculoskeletal disorders (MSDs) [1], particularly in the neck/shoulder (trapezius) region [2]. A proposed explanation for this is the lower muscle mass of females [3]—and consequently lower strength [4, 5]. Because of this lesser muscle mass, it is hypothesized that females completing the same task as males must work at a relatively higher percentage of their maximal capacity [6, 7]. Consequently, women may have an increased overload on their fewer and smaller muscle fibers [3, 8]. This hypothesis has been frequently advanced to explain sex differences in the incidence rates of work-related neck/shoulder MSDs [9].

However, it must also be accounted for that this higher muscle mass in males would lead to a higher weight of the body and the limbs that must be moved to ambulate and interact with the environment. Males also have longer arms [10]. These factors combined would imply that a greater torque is required to keep the arms lifted; in other words, that there is a greater anthropometric load (AL) for males. It is thus probable that differences in AL between individuals may contribute to differences in the development of fatigue in the neck and shoulder muscles responsible to stabilize the upper back when moving the arms to accomplish tasks of daily living and manual work. Following this logic, one would predict that the higher arm AL of men would that men would be at a higher biomechanical disadvantage. This disadvantage would likely result in a faster rate of fatigue when compared to females.

Fatigue has previously been identified as a work-related precursor to MSD development [11–13]. Muscular fatigue has been defined as a combination of increased perceived effort and decreased force generation capacity [14]. Repetitive movement and maintenance of static non-neutral postures requiring muscular effort, such as holding the upper limb at shoulder height in the sagittal plane, have been identified as risk factors for work-related MSDs [15]. This type of movement can lead to structural damage from occlusion of blood flow, with granulocyte plugging in the capillaries and subsequent accumulation of metabolites [16]. As repetitive movement has been associated with the development of fatigue which is, in turn, associated with the development of upper limb MSDs, the measurement of fatigue during repetitive upper limb movements can give us insights into MSD development [17].

In studies using measures of electromyographic (EMG) muscle activities, increased amplitudes have been associated with an increased motor unit recruitment, a strategy used to combat the onset of muscular fatigue while maintaining a given force [18]. In studies of upper limb repetitive tasks similar to those performed in sorting and in cashiers’ work, results have shown that fatigue leads to increased EMG amplitudes for the anterior deltoid and upper trapezius muscles [19]. The upper trapezius’s primary role is to stabilize the shoulder joint during upper limb movement, meaning it can be activated as a postural muscle for prolonged periods during upper limb repetitive movement. To allow for this, the oxidative capacity of the upper trapezius is high, with 66% of its muscle fibers being type I [20]. Conversely, the anterior deltoid’s primary role is to facilitate movement of the upper limb, especially in shoulder flexion. As such, the anterior deltoid must be able to generate high levels of force. This is reflected in the anaerobic nature of the muscle. It consequently has a low proportion of type I muscle fibers, roughly 33% [21].

Sex differences have been identified in the effects of fatigue on the variability of muscle activity [19, 22], while no clear sex differences in EMG amplitude changes have been found. In a fatiguing, repetitive pointing task, no sex differences in neck/shoulder amplitudes were found [19, 22]. However, in a repetitive, low-load, box folding task, females showed significantly higher increases in upper trapezius amplitudes [23]. While the latter study did not explicitly investigate the effect of fatigue, it may still imply that sex-specific motor control strategies were adopted by males and females. This posited sex difference in motor control strategy evolving during a fatiguing task, could produce differing levels of muscular fatigue during prolonged work, due to differing levels of muscle activation and responses to fatigue [24]. In sum, for whatever reason, one would expect to find sex differences in the development of fatigue during repetitive tasks.

However, in current biomechanical research, sex as a variable is often composed of many biological constructs. Through the inclusion of hypothesized constructs as covariates in statistical analyses, it may be possible to separate the effects of these constructs and the remaining sex effect. It is therefore important to identify constructs that contribute useful information about sex differences. One such relevant construct may be anthropometric differences. For example, research has identified a relationship between anthropometric differences and biceps brachii muscle activation during a fatiguing task [25]. Moderate to strong relationships have been found between maximal EMG amplitudes for the biceps brachii and skinfold thickness were found among both males and females. This result suggests that other anthropometric characteristics, such as the arm’s anthropometric load (AL), may play a role in explaining observed sex differences in fatigue-related EMG patterns (or the absence of such sex differences). While hypothetical mechanisms have been proposed to explain these sex differences, this area still needs further exploration [26]. Thus, the objective of this study was to determine if the arm’s AL, considered in this paper as a surrogate measure for the sex difference in limb anthropometrics of the arm, underlies the fatigue-related change in shoulder muscle activity in men and in women. We hypothesized that there would be sex differences in the fatigue-related changes in muscle activity following the performance of a shoulder fatiguing task, but that incorporating AL as a covariate would eliminate these sex differences.

## Materials and methods

### Subjects

A retrospective analysis was performed on data of 49 participants (25m/24f; Table 1) who were recruited using convenience sampling. Flyers were placed in the Jewish Rehabilitation Hospital research center and bulletin boards in the Department of Kinesiology and Physical Education at McGill University. Participants were excluded from the study if they had any history of mechanical upper limb and/or back pain/injury, or any conditions affecting balance, such as (but not limited to) neurological and vestibular conditions. The protocol, including the content of recruitment flyers, was approved by the ethics committee of the Center for Interdisciplinary Research in Rehabilitation of Greater Montreal. Part of the data has been previously analyzed in published articles [19, 27–31] but was never previously analyzed for the effects of AL.

**Table 1:** Descriptive statistics of participants

	*n*	Age (years)	Height (cm)	Body mass (kg)	BMI	AL (N m)	TTF (min)
**Male**	25	24.84 (± 1.20)	179.59 (± 1.71)	75.23 (± 1.72)	23.32 (± 0.39)	44.98 (± 2.74)	7.72 (± 0.66)
**Female**	24	24.04 (± 0.94)	164.45 (± 1.12)	58.30 (± 1.15)	21.57 (± 0.41)	19.87 (± 0.88)	9.04 (± 1.07)
**Overall**	49	24.45 (± 0.74)	172.18 (± 1.51)	66.94 (± 1.60)	22.47 (± 0.31)	32.68 (± 2.32)	8.37 (± 0.63)

Mean average values for each group are displayed with SE values included in parentheses

### Repetitive pointing task (RPT)

Participants completed a repetitive pointing task (RPT), as described by Fuller et al. [27]. Briefly, participants moved their dominant arm repetitively between two targets: one proximal (30% of arm length) and one distal (100% of arm length) aligned with the midline of the body and at shoulder height while standing. Each target provided auditory feedback when touched. A metronome was used to align these auditory signals when touching the target to a 1-Hz movement cadence. A mesh barrier was positioned under the participants’ elbow movement trajectory to ensure that the elbow remained elevated at shoulder height throughout the task. Participants self-reported their rating of perceived exertion (RPE) for the neck/shoulder region at the end of each minute using the Borg CR10 scale [32]. The task was terminated when participants either reported an RPE ≥ 8, verbally stated that they could not continue or could not keep pace with the metronome. Participants were unaware of these termination criteria.

### Measures

Upper limb and trunk kinematic data were acquired from the final 30 s of each minute at 120 Hz using 3-d optical motion capture (MX3 VICON, Oxford Metrics Ltd, Oxford, UK). A detailed description of the kinematic data acquisition and analysis has been reported previously [30]. In the current study, we only analyzed coordinates of the reflective markers that were placed on the upper arm (acromioclavicular joint, lateral epicondyle), forearm (medial and lateral styloid processes), and hand (second metacarpophalangeal joint, index fingertip) [27, 33].

Muscle activity was measured for the final 30 s of each minute using Ag-AgCl surface electromyography (EMG) electrodes (Ambu^TM^, Denmark). The centers of electrodes were placed 30 mm from one another. The electrodes were placed on the muscle belly of the anterior deltoid, identified as the point vertically below the lateral end of the clavicle, and the upper trapezius, identified as the midpoint between the acromion and the spinous process of the 7th cervical vertebrae (C7) [34]. Prior to electrode placement, the site was cleaned using alcohol, shaven, and slightly abraded using an abrasive gel to minimize skin impedance. Electrodes were oriented parallel to the muscle fibers. EMG data were collected at 1080 Hz, using a Telemyo 900 (Noraxon, USA) with an operating bandwidth of 10–350 Hz, an effective common mode rejection ratio of 130 dB DC, greater than 100 dB at 60 Hz, a minimum of 85 dB throughout the operating bandwidth and a fixed overall per-channel gain of 2000.

### Data analysis

EMG data was filtered using a 4th-order Butterworth filter, with a band-pass of 20–500 Hz. Root mean square (RMS) values were calculated from the data. Values were calculated over forward movement phases, where the arm moved towards the distal target. Thirteen to 15 forward movements were extracted, and the corresponding RMS values were averaged to represent no fatigue (NF) and fatigue-terminal (FT) data. Root mean square (RMS) [35] was used to quantify the amount of muscle activity. The signal from each EMG sensor was normalized as a percentage change from NF to FT using the equation:

$$ \mathrm{Normalized}\ \mathrm{RMS}\%\mathrm{Change}=\left(\mathrm{RMS}\ \mathrm{FT}-\mathrm{RMS}\ \mathrm{NF}\right)\div \mathrm{RMS}\ \mathrm{NF}\times 100 $$

Marker trajectories were low pass filtered at 15 Hz (zero lag, Butterworth, fourth order). AL was defined as the torque required to stabilize the upper limb at 90**°** shoulder flexion. Sex-specific body parameter equations were used to estimate this torque [36]. Briefly, sex-specific corrections were made to body segment parameter equations calculated from cadaver dissection [37] through hydrostatic weighing techniques. From these adapted equations, the weight of the upper limb segments was estimated as a percentage of total body mass. Lengths of the upper limb segments were estimated in one of two ways. For all participants who did not have 3D motion capture data collected during the RPT protocol (*n* = 28; 15m/13f), the length of the upper limb (measured from acromioclavicular joint to index fingertip) was used to estimate sex-specific, upper limb segment lengths (upper arm, forearm, and hand) [36]. All other participants’ (*n* = 21; 10m/11f) upper limb segment lengths were calculated using marker positions when participants were fully extended at end of the first reach in the NF trial, chosen to best replicate the manual arm length measurement. The upper arm segment length was calculated as the distance between acromioclavicular joint and lateral epicondyle markers. The forearm was calculated as the distance between the markers placed on the lateral epicondyle and the center point between the markers placed on the medial and lateral styloid processes. The hand segment length was calculated as the distance between the center point between the medial and lateral styloid processes markers, and index fingertip marker. Center of mass (CoM) position of each segment was estimated as a percentage of the total segment length, allowing for the position of the CoM of the entire upper limb to be estimated [36]. AL (N m) was calculated as weight of the upper limb multiplied by the distance of the CoM of the upper limb from the shoulder.

### Statistical analyses

*t* tests were run to determine statistical sex differences between the participant variables of height, body mass, body mass index (BMI), AL, age, and time to fatigue (TTF).

To allow comparisons between the classifications of AL and sex, AL groupings (low AL and high AL) were used to classify participants. As results using AL groupings were compared with sex groupings, sample sizes for low AL and high AL were kept consistent with the respective sex groupings (high AL: *n* = 25; low AL: *n* = 24).

The relationship between the fatigue effects on RMS values and sex or AL groupings were investigated. Pearson R correlations were run between AL and anterior deltoid (AD) RMS percentage change values, and between AL and upper trapezius (UT) RMS percentage change values. These correlations were run on the following groupings of participants: (1) all participants, (2) males, (3) females, (4) high AL group, and (5) low AL group.

Generalized estimating equations (GEE) were used to determine the effects of sex and muscle location (AD: *N* = 27; UT: *N* = 49) on RMS values. GEEs were run twice, once while including AL as a covariate and once without AL included as a covariate. This method of analysis was chosen over repeated measures ANOVA as it provides more power, is more robust against misidentified choice of a correlation matrix, helps estimate the average change per group, and is less restrictive in its assumptions [38, 39]. To determine significant differences between significant main effects and significant main interaction effects, estimated marginal means were calculated and pairwise comparisons were tested (Table 3). Hedges’ *g* effect sizes were computed from significant pairwise comparisons.

## Results

### Participant descriptives

Results of the one way *t* tests showed that males had significantly greater values for height (*F* = 50.92, *p* < 0.01, *η*^2^ = 0.52), body mass (*F* = 65.93, *p* < 0.01, *η*^2^ = 0.58), and BMI (*F* = 9.43, *p* < 0.01, *η*^2^ = 0.17), when compared to females. No significant age differences existed between males and females (*F* = 0.29, *p* = 0.59).

### AL

**Fig. 1:**
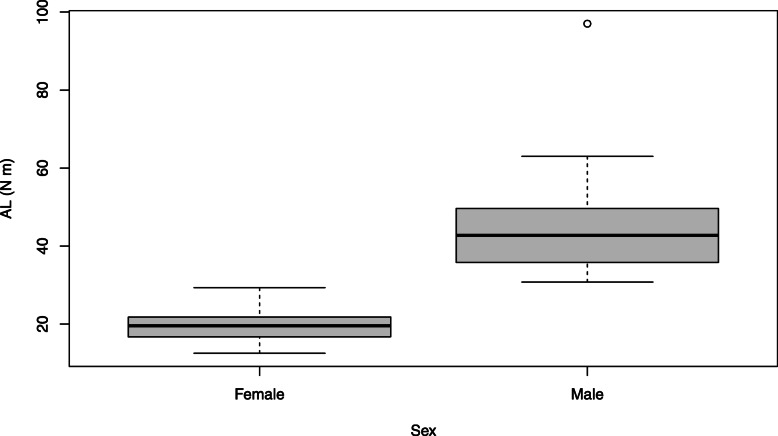
Boxplots depicting the AL values of females and males

Figure 1 displays the distribution of AL values of females and males included in this study. Females had significantly lower AL values than males (*F* = 74.42, *p* < 0.01, *η*^2^ = 0.78). In fact, all female participants had lower AL values than all male participants (females = 19.87 ± 0.88, males = 44.98 ± 2.74); therefore, when grouping participants into high or low AL groups, they were classified in the same way as sex. Subsequently, only results of sex groupings will be presented from this point onwards.

### TTF analysis

**Fig. 2:**
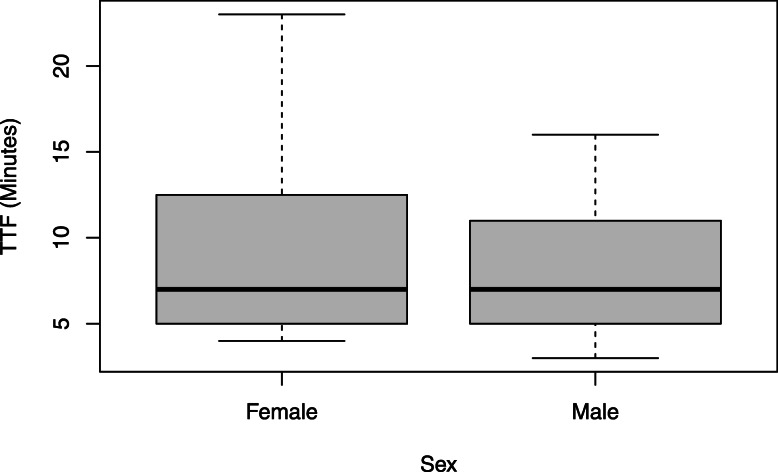
Boxplots depicting the TTF values of females and males

*t* tests run on participant descriptive statistics showed no significant differences in TTF between males and females (*F* = 1.12, *p* = 0.30). On average, males performed the task for 7.83 min, whereas females performed the task for 9.04 min (Table 1; Fig. 2).

### RMS

**Fig. 3:**
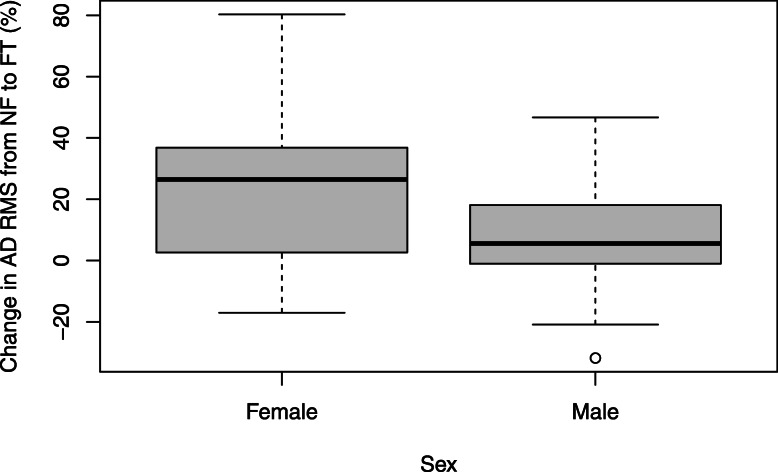
Boxplots depicting AD RMS % change values of females and males

**Fig. 4:**
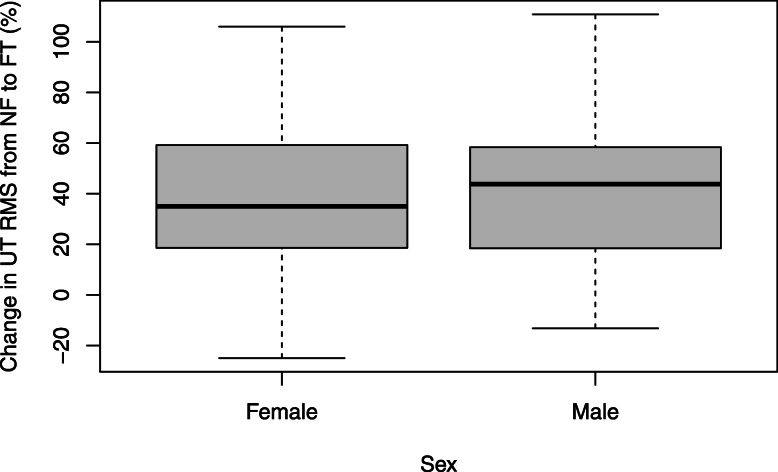
Boxplots depicting UT RMS % change values of females and males

Figure 3 displays the distribution of AD RMS percentage change values from NF to FT. Females had a 21.27% significantly higher mean change from NF to FT, when including AL as a covariate, compared to males (*p* = 0.03). There was no significant sex difference in UT RMS change with fatigue (Fig. 4) when including AL as a covariate (14.27% greater change in males, *p* = 0.31).

### Analysis of the relationship between RMS changes and AL

**Fig. 5:**
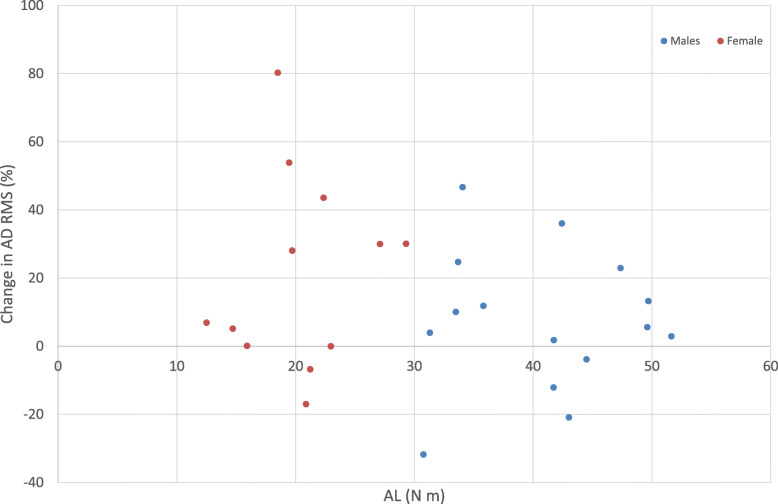
A scatter graph showing the relationship between change in AD RMS (%) and AL values

**Fig. 6:**
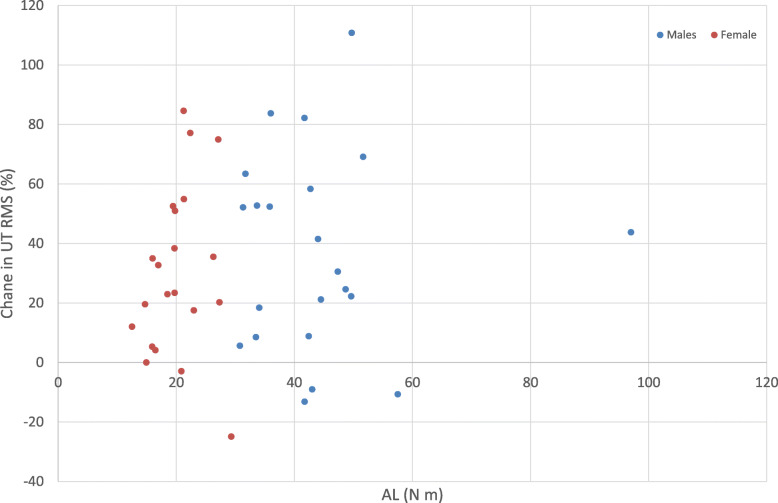
A scatter graph showing the relationship between change in UT RMS (%) and AL values

There were no relationships between AL and any percentage changes in AD (Fig. 5) or UT (Fig. 6) RMS values: whether the arm represented a heavy or light anthropometric load had no impact on the minute-1 to last-minute change in EMG RMS. In other words, when analyzing the whole group, the correlation between AL with percentage change in AD RMS and in UT RMS were *r* < 0.01, *p* = 0.99, and *r* = − 0.03, *p* = 0.84, respectively. In males, corresponding correlation coefficients were *r* = 0.29, *p* = 0.28, and *r* = 0.12, *p* = 0.57, respectively. For females, corresponding correlation coefficients were *r* = 0.33, *p* = 0.30, and *r* = 0.02, *p* = 0.92, respectively.

### GEE analysis

Results of the GEE analyses (Table 2) show a main effect of muscle. When including AL as a covariate, a significant main effect of sex was observed.

**Table 2:** Results of the GEE analysis for sex and muscle RMS

	Sex	Muscle	Sex × muscle	AL
With AL covariate	***χ***^**2**^**(1) = 4.17,** ***p*** = 0.04*	***χ***^**2**^**(1) = 8.48,** ***p*** < 0.01**	*χ*^2^(1) = 2.03, *p* = 0.15	*χ*^2^(1) = 2.12, *p* = 0.15
Without AL covariate	*χ*^2^(1) = 2.54, *p* = 0.11	***χ***^**2**^**(1) = 8.67,** ***p*** < 0.01**	*χ*^2^(1) = 2.01, *p* = 0.16	NA

*A main effect with significance set at *p* < 0.05

**A main effect with significance set at *p* < 0.01

The values in the parenthesis are the Wald Chi-square value and the corrected *p* values for RMS values and 95% confidence interval for difference for the pairwise comparisons.

Pairwise comparisons of the sex by muscle interaction (Table 3) displays significantly lower AD RMS for males compared to females (*p* = 0.03, *g* = 0.91), when including AL in the GEE model.

**Table 3:** Estimated marginal means (SE) and mean percentage difference in RMS from NF to FT

	Muscle	Male	Female	Mean difference	***p***
**With AL**	AD	7.10 (4.57)	28.36 (8.41)	− 21.27	0.03*
	UT	34.27 (6.78)	48.54 (10.30)	− 14.27	0.31
**Without AL**	AD	8.12 (5.11)	24.76 (7.58)	− 16.64	0.07
	UT	39.25 (6.26)	42.95 (7.67)	− 3.70	0.71

*A difference in means with significance set at *p* < 0.05

## Discussion

The main findings of our study were that AL did not group participants differently from sex in relation to our EMG variables, and no relationship was found between AL values and the percentage change of AD or UT RMS from NF to FT. However, when including AL as a covariate in the GEE model, a significant effect of sex was found. A significantly greater fatigue-related increase in AD RMS (but not in UT RMS) was observed for females compared to males. When AL was not included as a covariate, this sex effect disappeared. This suggests that, although AL is not a significant covariate on its own, it did improve the accuracy of the GEE model.

No significant differences were found in TTF between males and females (Fig. 2). This is consistent with previous findings in studies that analyzed sex differences using the same experimental task [19]. Moreover, when analyzing the dataset as a whole, or by sex, no significant relationships were found between percentage change of AD or UT RMS and AL values or between percentage change of UT RMS and AL values. This suggests that AL is not a useful independent predictor of fatigue-related changes in the RPT. Moreover, grouping participants using dichotomous values of AL, high or low, led to the same groupings as sex. Similarly, previous analysis of AD RMS changes from NF to FT in the RPT task found no sex difference [19]. However, the added value of taking AL into account lies in the possibility of using it as a continuous covariate, which leads to uncovering different muscle behaviors in both the male and female subgroups. Indeed, while AL may not provide novel information as a grouping variable, its usefulness as a covariate while modeling the effects of sex and muscle RMS was demonstrated. It is possible that AL as a covariate acts to reduce within-sex group variance, making it easier to observe between-group differences in the same variable as observed with AD.

When AL was included as a covariate, it could be seen that female AD muscles fatigued to a greater extent than males. This suggests that sex-specific factors independent of AL would explain this sex difference. For example, it has been established through muscle biopsies of the vastus lateralis and tibilais anterior that females have a greater proportion of type I muscle fibers compared to males [40–44]. Similarly, females have been shown to have smaller muscle fibers than males in the descending trapezius [8]. Type I muscle fibers are typically fibers with lower thresholds for activation and are therefore the first to be activated during tasks [45]. During repetitive movement, these low-threshold motor units likely remain active for the duration of the movement. Therefore, they are the first fibers to become fatigued, as proposed by the Cinderella hypothesis [46]. Even though this suggests that people with higher proportions of type 1 fibers would fatigue later, evidence from biopsy studies suggests that type 1 fibers are the target of injuries due to repetitive, low-intensity efforts, which has been proposed as one mechanism to explain the higher work-related injury prevalence of women assigned to such tasks [3].

Interestingly, although sex differences in AD fatigue were seen, no such differences were found in UT RMS values. During the RPT task, the AD is the prime mover, while the UT’s primary function is to stabilize the shoulder joint. Therefore, it seems logical that the AL of the upper limb, which needs to be overcome to produce the shoulder flexion motion and to maintain the glenohumeral joint flexed and the arm in an anterior position, would predominantly require activation of the AD, and thus that differences in AL would affect AD more than the UT.

As for the UT, previous research has indeed shown that the RPT specifically fatigues the UT [22, 28], and the present study corroborates this finding. Indeed, a greater fatiguing effect was observed, as shown by greater value for percentage increase in UT values during the final minute, compared to the AD values (Table 3). Sex differences in motor control strategies have also been suggested from sex differences in coactivation of shoulder muscles [47]. When stabilizing the arm through isometric contractions in the sagittal plane, comparable to the isometric contractions required to stabilize the arm in the RPT, females showed a greater activation of the descending trapezius (or UT). This contraction of non-agonistic muscles is ultimately less efficient for task performance. In the RPT, this may be reflected as a greater fatiguing effect in the female group for the UT and chronically, the repetitive overloading of these muscle fibers may be related to an increased incidence of neck/shoulder MSDs in females [3]. However, the fact that there were no sex differences in UT in the present study suggests that other characteristics of trapezius muscle activation, such as shared activation between different portions of the trapezius [22, 23], may contribute to the absence of sex differences in results involving the UT in the present study.

Sex differences in shoulder control and coordination may also be seen through the relative intensities of contractions. Females have been shown to work at a higher relative intensity compared to males when completing the same fatiguing, upper limb task [48]. Working at a relatively higher intensity over a chronic period could also contribute to the observed sex difference in incidence rates of upper limb MSDs. To investigate this further, a study examined the fatiguing effects of a sustained, sub-maximal, elbow flexion task, when matching sex groupings for maximal wrist extensor strength [49]. The strength-matched groups displayed similar decreases in maximal strength after the fatiguing task, but differences in EMG activity existed between sexes. At the end of the fatiguing task, females showed a greater increase in EMG bursts. This suggests that sex-specific differences exist in the motor control strategies implemented to continue performing a task while fatigued [3, 19]. In the present study, pairwise comparisons of the interaction effects between sex and muscle, when including AL as a covariate, revealed a significantly higher value for percentage change in AD RMS amplitude from NF to FT in females compared to males. These results suggest that, even when controlling or matching participants for significant covariates such as AL or strength, sex-specific differences in motor control strategies are still observed. This strengthens the rationale that sex-specific motor control strategies exist in upper limb fatiguing tasks.

Finally, it should be noted that although we used anthropometric load as our independent anthropometric variable, there are other anthropometric characteristics that are known to distinguish upper body structure of males and females. As such, skinfold thickness has been investigated in relation to EMG values [25]. It was found that skinfold thickness, regardless of sex, had a significant relationship with EMG of the biceps brachii during a fatiguing task. This suggests that different anthropometric factors, such as skinfold but perhaps also others, could play roles in explaining findings of studies on sex-specific mechanisms of fatigue. Future studies could aim to model the relative weights and ratios of the most salient anthropometric characteristics in order to more accurately estimate their impact on sex differences in mechanisms of upper limb fatigue.

Limitations and assumptions made during the protocol may reduce the external and internal validity of the project. Due to the retrospective nature of this study, EMG signals were normalized as a percentage change from NF to FT conditions. This may lead to some inconsistencies in fatigue rates between individuals. Other normalization techniques, such as normalizing the signal to a reference maximal voluntary contraction of the muscle, could be utilized in future research. That said, although MVC is a widely used technique for normalization, MVCs of healthy individuals, can be 20–40% less than those achieved after a practice session [50]. Moreover, data normalization techniques, particularly those using MVC, have been shown to affect observed sex differences in EMG. In other words, normalizing to MVC may artificially affect sex differences in EMG recorded during sub-maximal tasks [51]. The torque required to stabilize the arm at 90° was estimated, and underlying assumptions for this estimation were made. Sex-adjusted cadaver data [36, 37] was used to estimate locations of the CoM for each segment and consequently the upper limb. Individual differences in the distribution of mass would therefore create some level of inaccuracy in the individual AL values. It is unknown if the shape, volume, and, as a result, location of the arm CoM is the same for men as for women. Similarly, AL was calculated based on the length of the limb segments. It should be acknowledged that other aspects of the AL load of the limb, such as volume of the arm, were not estimated. This analysis of such dimensions may allow for more accurate analysis of the role of AL in sex differences for the studied task. Future research could incorporate DEXA scans for each participant to more accurately measure the distribution of mass for each segment and gain a more accurate representation of individual CoM [52]. Volumes of the arm segments could also be measured, allowing for advanced equations for AL to be theorized and utilized. All participants fulfilled the termination criteria for the RPT through a self-reported score of 8+ on the Borg CR-10 scale. Participants were instructed to rate their neck-shoulder exertion, and made no reference to pain. Since no scale that specifically asked about perception of pain was used in the current study, our interpretations of these results are limited to that of exertion. It should be noted, however, that previous research has investigated this link between pain and exertion in a similar task [53].

## Perspectives and significance

The results of this study imply that anthropometric variables may play an important role in the analysis of sex effects. The current body of published research suggests anthropometric variables such as strength [49] and skinfold thickness [25] have parameter specific results when investigating sex effects. In the current study, the use of AL as a covariate allowed for sex-specific differences to be separated from differences in the anthropometrics of participants. This ultimately allowed for a more valid estimation of the physical factors underlying sex differences in mechanisms of upper limb fatigue. Future research may attempt to use other anthropometric factors to separate sex differences from individual anthropometric differences.

## Conclusion

When including AL as a covariate, a significant main effect of sex was found on change in muscle activity amplitude with fatigue. This was not observed when AL was removed from the GEE model. The higher rate of fatigue for the AD muscle in females is likely due to sex differences in muscle fiber composition, and thus in the thresholds of activation for these fibers. This study raises the importance of the exploration of anthropometric variables, such as AL, to better understand the origin of sex-specific mechanisms of muscle fatigue, and, ultimately, of work-related injuries.

## Data Availability

The datasets used and/or analyzed during the current study are available from the corresponding author on reasonable request.
